# Cocrystal Formation of Betulinic Acid and Ascorbic Acid: Synthesis, Physico-Chemical Assessment, Antioxidant, and Antiproliferative Activity

**DOI:** 10.3389/fchem.2019.00092

**Published:** 2019-02-21

**Authors:** Mirela Nicolov, Roxana M. Ghiulai, Mirela Voicu, Marius Mioc, Adina Octavia Duse, Roxana Roman, Rita Ambrus, Istvan Zupko, Elena Alina Moaca, Dorina E. Coricovac, Claudia Farcas, Roxana Marcela Racoviceanu, Corina Danciu, Cristina-Adriana Dehelean, Codruta Soica

**Affiliations:** ^1^Faculty of Pharmacy, Victor Babes University of Medicine and Pharmacy, Timisoara, Romania; ^2^Faculty of Medicine, Victor Babes University of Medicine and Pharmacy, Timisoara, Romania; ^3^Faculty of Physics, West University of Timisoara, Timisoara, Romania; ^4^Institute of Pharmaceutical Technology and Regulatory Affairs, University of Szeged, Szeged, Hungary; ^5^Department of Pharmacodynamics and Biopharmacy, University of Szeged, Szeged, Hungary

**Keywords:** cocrystal, betulinic acid, vitamin C, antioxidant activity, antiproliferative activity

## Abstract

Betulinic acid (BA) was demonstrated to be a very promising anticancer agent against various tumor cell lines such as breast, colon, lung, and brain. Despite its strong cytotoxic effect, betulinic acid exhibits low water solubility, feature that is reflected in its poor bioavailability. To overcome these drawbacks, numerous strategies were conducted to improve its physicochemical and pharmacokinetic profile, among which cocrystalization emerged as a promising approach. Thus, our work consisted in obtaining slowly grown cocrystals of BA and ascorbic acid (BA+VitC) in isopropyl alcohol obtained in a hydrothermal experiment. The newly formed cocrystals were characterized by physico-chemical methods such asSEM, DSC, XRPD, and FT-IR spectroscopy demonstrating BA+VitC cocrystal formation while their antioxidant activity revealed an additive antioxidant effect. To investigate the biological effect, BA+VitC cocrystals were tested on HaCat (immortalized human keratinocytes), B164A5 and B16F0 (murine melanoma), MCF7 and MDA-MB-231 (human breast cancer), and HeLa (cervical cancer) cell lines. Results of BA upon the tested tumor cell lines, after co-crystallization with vitamin C, indicated a superior cytotoxic effect with the preservation of a good selectivity index assumably due to an improved BA water solubility and consequently an optimized bioavailability.

## Introduction

The field of pharmaceutical formulations is similar to a living organism, constantly changing, and adapting to new therapeutic needs. Numerous newly reported active chemical compounds exhibit low water solubility and dissolution rate which may correlate to poor bioavailability, especially via oral administration (Gadade et al., 2016). Among the current options to mitigate these flaws (Prasad et al., 2012), crystal engineering provides several possibilities to develop single- or multi-component alterations of an active compound, including the synthesis of pharmaceutical cocrystals (Pathak et al., 2013). Cocrystals are crystalline complexes of active/neutral compounds that form a unique crystalline lattice through non-covalent bonds, in particular hydrogen bonds; the main benefit associated with cocrystallization consists in the preservation of the intrinsic pharmacological properties of the active ingredients while the physicochemical profile (i.e., melting point, solubility, dissolution, etc.) changes (Prasad et al., 2012). Drug-drug cocrystals represent a promising line of research in light of the fact that combined therapies are frequently prescribed for the effective treatment of numerous pathologies (Sekhon, 2012); cocrystals of multiple active compounds might overcome the drawbacks of conventionally combined drugs.

Betulinic acid [BA, 3β-hydroxy-20(19)-lupan-28-oic acid] is a pentacyclictriterpene of lupan skeleton with a wide range of pharmacological activities such as: anti-inflammatory, antitumor (Faujan et al., 2009), anti-angiogenic (Mukherjee et al., 2004), anti-viral (Baltina et al., 2003), and antiplasmodial (Ziegler et al., 2004); its first anticancer effect against melanoma was reported by Pisha et al. (1995). The antitumor activity of betulinic acid is currently known to be selectively exerted on numerous tumor cell lines (breast, colon, lung, brain) mainly by apoptosis induction (Fulda and Kroemer, 2009). The favorable therapeutic index suggests betulinic acid as promising antitumor agent (Pisha et al., 1995). It also possesses significant antioxidant properties as revealed by Peng et al. (2015).

Betulinic acid is characterized by high hydrophobicity and limited aqueous solubility leading to poor bioavailability; therefore, it qualifies as candidate for cocrystallization in order to achieve superior pharmacokinetic properties.

Ascorbic acid, vitamin C, (2R)-2-[(1S)-1,2-dihydroxyethyl]-3,4-dihydroxy-2H-furan-5-one (VitC), is a natural highlywater-soluble vitamin which acts as a potent reducing and antioxidant agent; its biomedical uses reside in fighting bacterial infections, in detoxifying reactions, and in the formation of collagen in fibrous tissue, teeth, bones, connective tissue, skin, and capillaries. VitC was previously used as cocrystal former for several nutraceuticals in a patent issued in 2008 for the purpose of providing improved properties, in particular as oral formulations (Zaworotko et al., 2008). It also acted as cocrystal former for several zwitterion structures (i.e., sarcosine, nicotinic acid, betaine) leading to cocrystals that exhibit carboxylate-hydroxyl supramolecular heterosynthons (based on intermolecular hydrogen bonds) (Kavuru et al., 2010).

Up until recently, a significant number of computational methods that can predict new cocrystal formation from various structures have been developed. Such an approach consists in the determination of molecular electrostatic potential surfaces (MEPS) for molecular complementarity assessment, as well as the assessment of potential cocrystal formation based on an electrostatic model described by intramolecular interactions as a set of specific contact interaction points (SSIPs) on the molecular surfaces (Karagianni and Malamatari, 2018).

A synergistic antioxidant effect of betulinic acid and ascorbic acid mixture was described by Adesanwo et al. (2013) who suggested the ascorbic acid-mediated chain-breaking electron transfer to DPPH (2,2-diphenyl-1-picrylhydrazyl) followed by ascorbic acid regeneration due to proton transfer from betulinic acid, thus leading to a resonance stabilized betulinic acid radical (Adesanwo et al., 2013).

This paper is the first approach to obtain and characterize slowly grown cocrystals of betulinic acid and ascorbic acid, using as solvent isopropyl alcohol. Given the already reported significant antioxidant activity of both compounds and that a Cambridge data base search was made and the a BA+VitC cocrystal has yet to be reported, we also aimed to assess the synergistic/additive result of cocrystallization in terms of this particular biological effect. To the best of our knowledge, no previous studies of betulinic acid cocrystals were reported. In addition, the biological *in vitro* activity of the cocrystals was tested on both normal and cancer cell lines.

## Materials and Methods

### Materials

Betulinic acid (99%), L-ascorbic acid (99.7–100.5%), 2,2-diphenyl-1-picrylhydrazyl (DPPH) were purchased from Sigma-Aldrich, Germany; isopropyl alcohol and ethanol 96% (v/v) from Chemical Company SA, Iasi, Romania. All substances were used without further purification.

### Gas Phase *ab initio* DFT Calculations for BA-VitC Cocrystal Formation

This method is based on the calculation of gas phase MEP for the proposed structures, followed by the conversion of local MEP maxima and minima into SSIPs, α and β, that describe the potential H-bond interaction sites (Musumeci et al., 2011; Grecu et al., 2013). Structure geometry optimization and DFT calculations were employed by using the GAMESS software while MEPS were generated using MacMolPlt and Avogadro. Geometry optimization and MEP for both structures were achieved by *ab initio* gas phase DFT calculations, at the B3LYP 6-31G(d) level of theory. Local MEP maxima and minima were converted in SSIPs using the following equations (Equations 1, 2) (Musumeci et al., 2011);

(1) $$\alpha =0.0000162MEP_{max}^{2}+0.00962MEP_{max}$$

(2) $$\beta =0.000146MEP_{min}^{2}-0.00930MEP_{min},$$

where:

MEP_*max*_ and MEP_*min*_ represent local maxima and minima (energy values were given in Hartrees)

Calculated SSIPs were paired for the pure structures and the cocrystal as follows: the highest α values interact with the highest β values, the second highest α value interact with the second highest β and so on. After α/β pairing, the total interaction site energy for the pure solids and cocrystal was estimated using Equation (3) (Musumeci et al., 2011). Energy values were converted from Hartrees into kJ/mol.

(3) $$E=-\sum_{ij}\alpha i\text{​​}\beta \text{​​ j}\;$$

The calculated energy difference between the interaction site pairing energies of the cocrystal and the two pure forms (Equation 4) provides a probability measure for a cocrystal formation (1:1) based on the assumption that within the cocrystal more favorable interactions will be formed.

(4) $$\Delta E=E_{ccry}-xE_{a}-yE_{b}$$

where:

E_ccry_ is the calculated interaction energy of the cocrystal,

E_a_ and E_b_ are the calculated interaction energies for the pure structures, and

x,y represent the molar ratio of the two structures within the cocrystal.

### Synthesis of BA+VitC Cocrystals From Isopropyl Alcohol

30.3 mg BA (MW 456.71 g/mole) and 11.7 mg VitC (MW 176.12 g/mole) were solubilized in 1:1 molar ratio using 4 ml isopropyl alcohol. The mixture was heated at 55°C and then allowed to cool slowly at room temperature, protected from light. The formation of the first crystals was recorded after 10 days.

### Scanning Electron Microscopy (SEM)

The morphology of the sample was investigated by SEM–scanning electron microscopy (Hitachi S4700, Hitachi Scientific Ltd., Tokyo, Japan) at 10 kV. The samples were gold-palladium coated (90 s) with a sputter coater (Bio-Rad SC 502, VG Microtech, Uckfield, UK) using an electric potential of 2.0 kV at 10 mA for 10 min. The air pressure was 1.3–13.0 mPa.

### Differential Scanning Calorimetry (DSC)

The thermal response of each product was measured using a differential scanning calorimeter (Mettler Toledo TG 821^e^ DSC Mettler Inc., Schwerzenbach, Switzerland). About 3–5 mg of powder was precisely weighed into DSC sample pans which were hermetically sealed and lid pierced. Samples were measured in the temperature range of 25–350°C at a heating rate of 10°C/min under constant argon flow of 150 ml/min. Data analysis was performed using the STAR^e^ software.

### X-Ray Powder Diffraction (XRPD)

The XRPD measurement was carried out with a BRUKER D8 Advance X-ray powder diffractometer (Bruker AXS GmbH, Karlsruhe, Germany) with Cu K λI radiation (λ = 1.5406 Å) and a VÅNTEC-1 detector. The powder samples were loaded in contact with a plane quartz glass sample slide with an etched square, and measured. Samples were scanned at 40 kV and 40 mA. The angular range was 3–40° 2θ, at a step time of 0.1 s and a step size of 0.007°. All manipulations, including Kα2-stripping, background removal, and smoothing of the area under the peaks of the diffractograms were performed using the DIFFRACTPLUS EVA software.

Rietveld analysis on the obtained X-ray diffraction pattern, was carried out using the MAUD software. The refinement was carried out using available structural crystallographic information of the two constitutive phases, namely BA and VitC.

### Fourier-Transform Infra-Red Spectroscopy (FTIR)

FTIR spectra were recorded on a JASCO 670+ instrument after KBr pelleting. The data were collected in 4,000–400 cm^−1^ spectral range. Spectra were built up after a number of 24 acquisitions for each spectrum, with a resolution of 2 cm^−1^.

### Antioxidant Activity Assay

An amount of cocrystal material was dissolved in 1 ml isopropanol in order to obtain a final concentration of 1.04 mg/ml. The antioxidant activity (AOA) was evaluated by DPPH radical scavenging assay, which was originally described by Blois (1958). Briefly, 1 mmol·L^−1^ solution of DPPH was prepared and stored at 4°C, in the dark, and was used as a standard antioxidant stock solution. A reference solution of 0.167 mmol·L^−1^ascorbic acid in ethanol 96% (v/v)was also prepared; 0.5 mL of the cocrystal and ascorbic acid solutions, were each added to a 2.5 mL diluted stock solution of DPPH (2 mL ethanol 96% (v/v) + 0.5 mL DPPH 1 mM). The mixture was then analyzed using an Uvi Line 9400 Spectrophotometer from SI Analytics at 516 nm for 20 min.

Antioxidant activity was calculated using the following equation:

(5) $$AOA\;\left(\%\right)=100-\frac{A_{516_{\left(sample\right)}}}{A_{516_{\left(DPPH\right)}}}\cdot 100$$

where:

AOA = antioxidant activity [%];

*A*_516(*sample*)_ = absorbance of the sample measured at 516 nm at a specific time;

*A*_516(*DPPH*)_ = absorbance of the standard solution measured at 516 nm (without sample).

### Cell Culture

HaCat (immortalized human keratinocytes), B164A5, and B16F0 (murine melanoma) cells were cultured in specific culture medium–Dulbecco's modified Eagle Medium (DMEM) with high glucose (4.5 g L^−1^), L-glutamine and sodium bicarbonate, supplemented with 100 U mL^−1^ penicillin, 100 μg mL^−1^ streptomycin, and 10% fetal bovine serum (FBS). The number of cells used in the experiments was determined in the presence of Trypan blue using a Neubauer counting chamber (Coricovac et al., 2017). HaCat and B16F0 cells were purchased from ATCC (American Type Cell Collection) and B164A5 from Sigma-Aldrich Chemie GmbH (Munich, Germany).

Human breast cancer (MCF-7, MDA-MB-231) and cervical (HeLa) cell lines were purchased from European Collection of Cell Cultures (Salisbury, UK). The cells were grown in Eagle's Minimum Essential Medium (EMEM) supplemented with 10% heat-inactivated fetal bovine serum (FBS), 1% non-essential aminoacids, and 1% antibiotic-antimycotic mixture. All media and supplements were obtained from Lonza Group Ltd. (Basel, Switzerland). The cells were cultured in a humidified atmosphere with 5% CO_2_ at 37°C and were passaged at every two-three days.

### Determination of Cell Viability

The antiproliferative activities of the tested substances were determined by standard MTT (3-(4,5-dimethylthiazol-2-yl)-2,5-diphenyltetrazolium bromide) (Mosmann, 1984) and Alamar blue assays. **MTT:** briefly, cells were plated into 96-well plates at a density of 5,000 cells/well and pre-incubated overnight. After incubation for 72 h with test compounds (BA+VitC cocrystal−3, 10, and 30 μM), 20 μl of MTT solution was added and the plates were incubated for another 4 h. The formed formazan crystals were dissolved in 100 μl dimethyl sulfoxide and the absorbance was determined at 545 nm. Wells with untreated cells were utilized as control.

**Alamar blue:** The cells (1 × 10^4^/200 μL medium/well) were seeded in a 96-well plate and allowed to attach. After proper confluence was reached, cells were incubated with different concentrations (3, 10, and 30 μM) of the BA+VitC cocrystal for 48 and 72 h. After the incubation period, the Alamar blue reagent was added to each well (20 μL/well). The plates were incubated for 3 h at 37°C and the absorbance of each well was measured using a xMark™ Microplate Spectrophotometer (Biorad) at 570 and 600 nm (reference) wavelengths. Cell viability was calculated according to the formula described in our previous articles (Soica et al., 2014b).

For both assays, MTT and Alamar Blue, DMSO (dimethyl sulfoxide) was used as negative control. Even at the highest concentration tested−30 μM, the percentage of DMSO in the growth medium was 0.03%, percentage that is considered non-toxic for cells.

## Results and Discussions

Cocrystallization emerged as a successful procedure to modulate the properties of solid active ingredients for the purpose of improving drug delivery as well as drug manufacture; cocrystals not only provide optimized physicochemical (i.e., solubility, stability) and mechanical properties of the active drugs but they also offer the possibility of combined therapies that come along with intellectual property opportunities (Sun, 2012). In addition, multidrug cocrystals may exhibit synergistic or additive pharmacological effects. The design of possible cocrystals involves the evaluation of potential non-covalent and non-ionic intermolecular interactions between two or several active ingredients; in general, cocrystal formers bear functional groups that have the ability to form hydrogen bonds (Du et al., 2016).

Betulinic acid was identified as a very promising anticancer agent and included in the Rapid Access to Intervention Development (RAID) NCI program; however, its low water solubility and permeability cause a poor bioavailability which, despite its oral activity, imposes the necessity of large oral doses (Godugu et al., 2014). BA solubility and permeability properties may assimilate it as a BCS class IV compound which generally does not reach market development (Chavda et al., 2010). Previous studies reported various attempts to solve BA physicochemical and pharmacokinetic drawbacks through specific formulations: (1) cyclodextrin complexation (Soica et al., 2014a), (2) nanoemulsion (Dehelean et al., 2013), (3) liposomes (Mullauer et al., 2011), carbon nanotubes (Tan et al., 2014), (4) polymer micelles (Das et al., 2016), or conjugates (Lomkova et al., 2016). However, such formulations can be problematic in terms of large scale production as well as storage (Sanphui et al., 2015); therefore, crystal engineering might offer a superior formulation alternative.

In designing potential cocrystals, a key point is the possibility of intermolecular interactions between components, in particular hydrogen bonds, that may trigger the molecular assembly and determine the supramolecular architecture (Venugopalaiah et al., 2016). The formation of a cocrystal depends upon the existence of a coformer that dramatically influences the cocrystal properties, mainly solubility, and dissolution (Tomaszewska et al., 2013). To date, the selection of a suitable coformer is based on several predictive criteria (Schultheiss and Newman, 2009); however, the formation of a real cocrystal can be validated only through valid experimental procedures. The most frequently used coformers are compounds bearing functional groups such as carboxyle, amide, hydroxyle, or amino groups (Jain et al., 2015). We assume that this new compound BA+VitC in isopropanol monosolvate can be linked together by the carboxylic bond.

Vitamin C was previously involved as coformer in the formation of cocrystals (Kovac-Besovic et al., 2009; Meepripruk et al., 2016); moreover, its antioxidant activity could synergistically combine with the intrinsic antioxidant effect of BA thus leading to a more soluble and higher biologically active cocrystal. Another key element in cocrystallization is the selection of solvents which depends on the solubility of both drug and coformer and strongly influences the stoichiometry of the resulting cocrystal (Leyssens et al., 2014).

### Gas Phase *ab initio* DFT Calculations for BA-VitC Cocrystal Formation

Calculated site pairing energy values for the pure forms of the two structures and the expected cocrystal (1:1 ratio) are depicted in Table 1. The obtained ΔE value (−1.79 kJ/mol) suggests that in the new cocrystal more favorable interactions may be formed. Other previously reported ΔE values for confirmed 1:1 ratio cocrystals, such as: caffeine:acetic acid (ΔE = −1), caffeine:adipic acid (ΔE = −3), caffeine:sulfacetamide (ΔE = −2), caffeine:sulfaproxyline (ΔE = −1) (Musumeci et al., 2011), fall in the same range as our reported value. We can therefore conclude that a BA-VitC 1:1 ratio cocrystal was formed as a result of our crystallization process.

**Table 1:** Calculated site pairing energy values for the pure forms of the two structures and the expected cocrystal (1:1 ratio).

**Compound**	**Calculated site pairing energy –α_i_β_j_ (kj/mol)**	**Calculated ΔE (kj/mol)**
VitC	−22.76	
BA	−20.71	−1.79
VitC+BA proposed cocrystal (1:1)	−45.26	

### Scanning Electron Microscopy (SEM) Analysis

**Figure 1:**
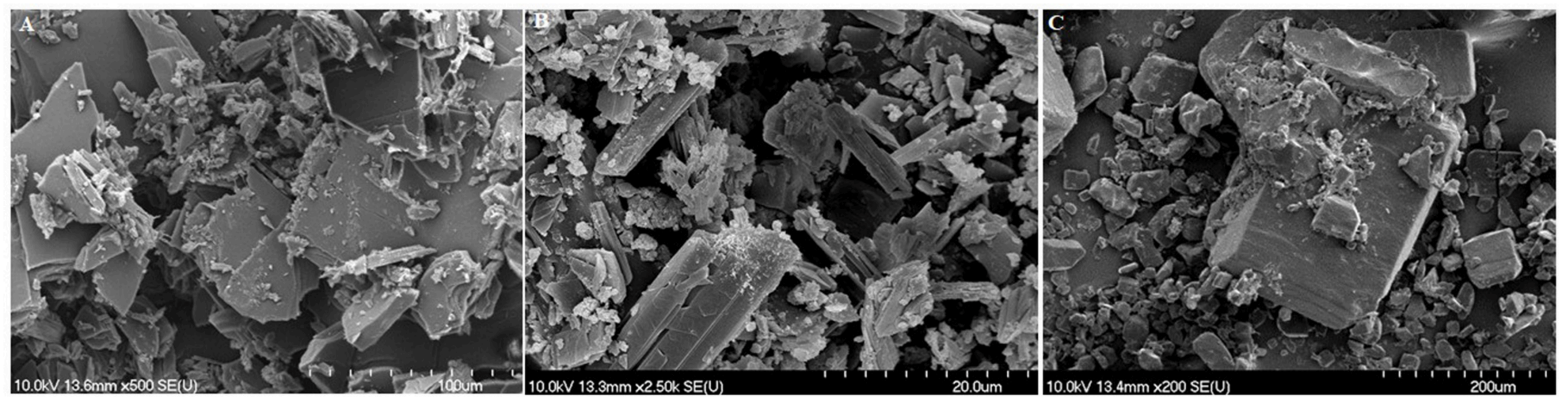
SEM images of BA+VitC cocrystals **(A)**, pure BA **(B)**, pure VitC **(C)**.

SEM images of pure BA, pure VitC, and of their 1:1 cocrystal resulted from isopropanol solution are depicted in Figure 1. The scanning electron microscopy images show morphological differences between the pure ingredients and their cocrystallization product, thus indicating the formation of a new compound, formed through layer by layer deposition.

### X-Ray Powder Diffraction Pattern

In order to provide an exhaustive structural characterization at atomic level, a single crystal structure would be needed; however, this single crystal might not be representative for the polycrystalline product used as source. Moreover, single crystal analysis requires a large enough and flawless crystal which is difficult to achieve. In light of these facts, the more readily available X-ray powder diffraction pattern (XRPD) is generally used to assess a powder crystalline material in terms of crystal structure, chemical composition, and physical properties.

**Figure 2:**
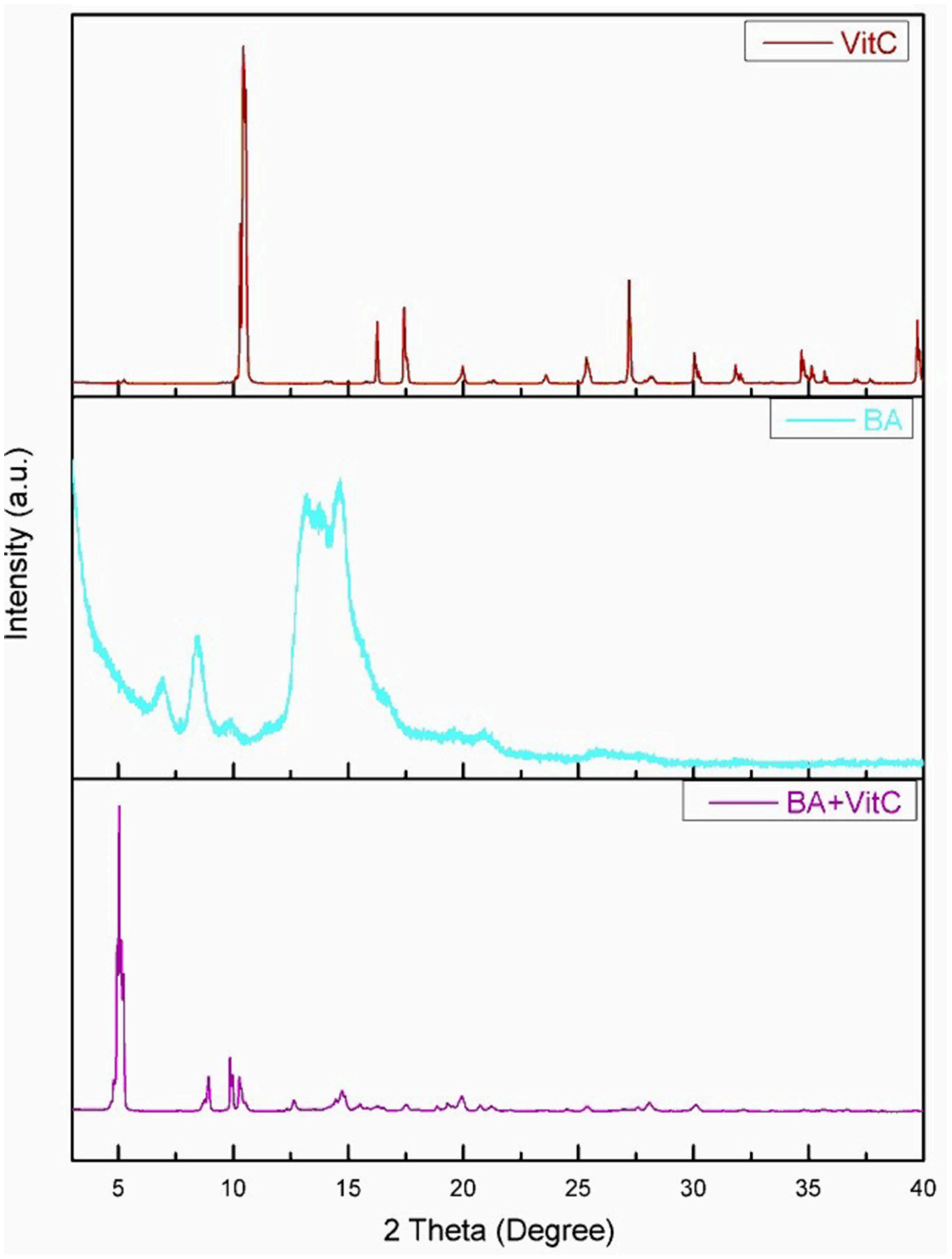
XRPD spectra for BA, VitC, and BA+VitC cocrystal.

The XRPD spectra forBA, VitC, and 1:1 BA+VitC cocrystal, respectively, are presented in Figure 2. The BA+VitC profile exhibits significant differences as compared to the ones recorded for the pure compounds, thus suggesting the formation of the cocrystal; in case of a simple physical mixture, the XRPD spectrum of the sample would be the sum of the diffraction peaks recorded for the pure compounds (BA and VitC) lacking any new diffraction pattern.

The new formed peaks of the BA+VitC cocrystals are present at 2θ, the most intense peaks are depicted at the following values: 5.04/8.82/8.94/9.86.The XRPD of the pure compounds revealed the existence of crystal lattices; in the cocrystal diffractogram, the peaks of starting materials are absent thus indicating the formation of a new crystalline phase and supporting the formation of an actual cocrystal.

**Figure 3:**
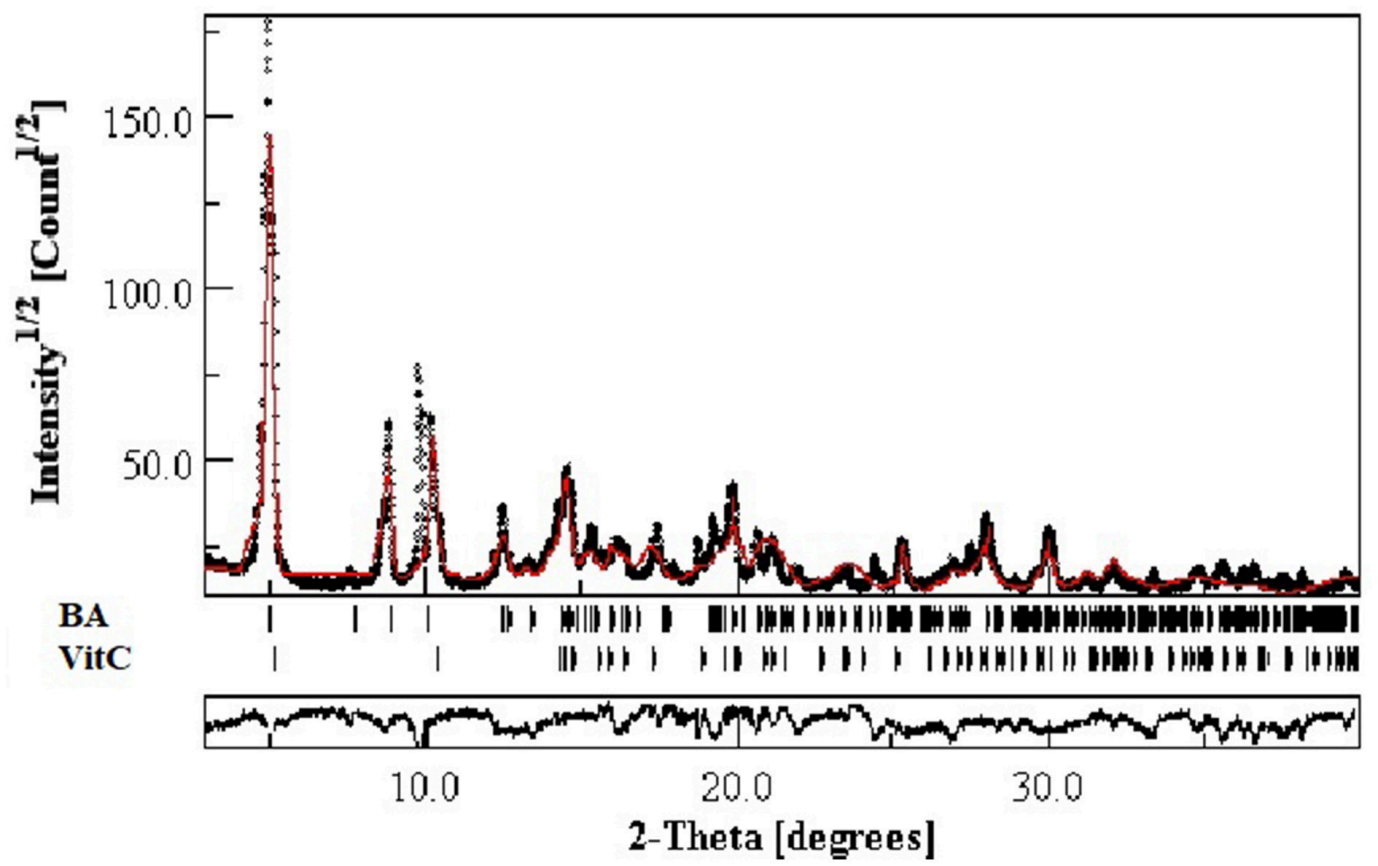
Experimental XRPD pattern of proposed BA+VitC cocrystal compared with Rietveld refined profile (red continuous line).

Rietveld refinement of experimental XRPD pattern corresponding to the proposed BA+VitC cocrystal is depicted in Figure 3. The values obtained as a result of quantitative analysis indicate the following composition, BA weight (%): 64.2742 and VitC weight (%) 35.72522, although calculated analysis parameters such as the GOF (goodness of fit) = 2.24%, Rwp = 11.24%, and Rexp = 4.65% are slightly above the values that ascertain a good fit, as indicated by the software developer. Nevertheless, the refined profile, calculated according to the corresponding data of the two phases, as seen in Figure 3, shows a visual overall good fit. The high values of the analysis parameters, mentioned above and a visual analysis of the refined profile line, revealing that some peaks are not well-fitted, could indicate polycrystalline forms (excess crystal forms of the components) present in the analyzed powder. Taking all of this in consideration and the fact that there is little crystallographic structural information regarding the two phases (BA and VitC), for obtaining a better fit, we can conclude that the formation of the cocrystal was achieved but proposing a structure for the new cocrystal is very difficult and it will constitute a research purpose for future studies.

### Differential Scanning Calorimetry (DSC) Analysis

DSC is a thermal analysis that quantifies the difference between the heat amounts required to increase the temperature of the analyzed sample and the reference, respectively, as a function of temperature or time.

**Figure 4:**
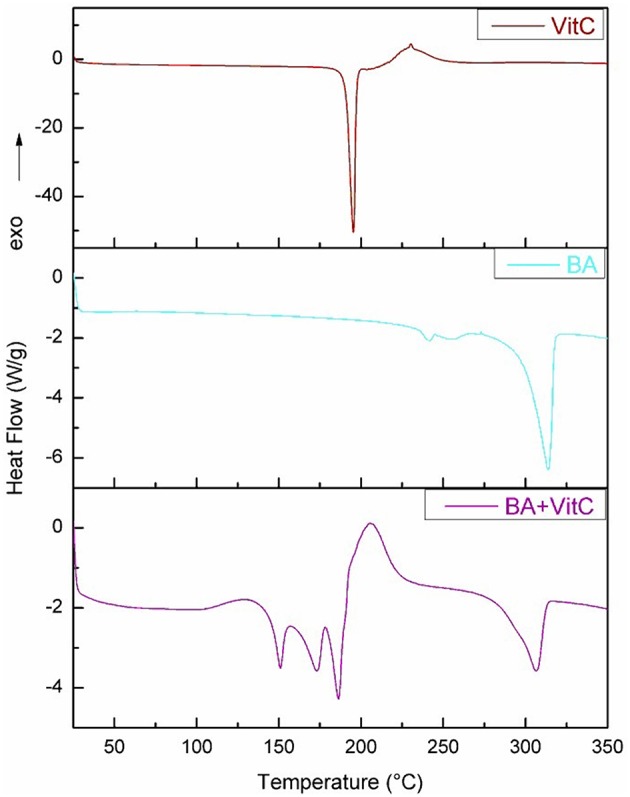
DSC curves forBA, VitC, and BA+VitC.

Characteristic and comparable thermograms were recorded for both pure ingredients BA and VitC and for their cocrystal as well. The DSC curves for BA, VitC, and BA+VitC cocrystal are presented in Figure 4.

For BA two small endothermic peaks at 241.23 and 255.56°C and a strong endothermic peak at 313.63°C can be noticed, corresponding to the melting of the compound accompanied by decomposition. For VitC a sharp endothermic peak is present around 192.8°C, attributed to the melting point of the compound, followed by a small exothermic peak correlated with the beginning of chemical degradation. For BA+VitC cocrystal: 3 endothermic peaks appear at 150.85, 173.17, and 186.14°C, respectively, followed by an exothermic process that may indicate the formation of a new structure that starts to decompose at 291.39°C; the process is finished above 300°C. The W-shaped thermogram (Yamashita et al., 2013; Stoler and Warner, 2015) such as the one obtained for BA+VitC proved to be characteristic for the cocrystals, different from the V-shaped specific for the eutectic mixtures.

### Fourier-Transform Infra-Red Spectroscopy (FT-IR) Analysis

**Figure 5:**
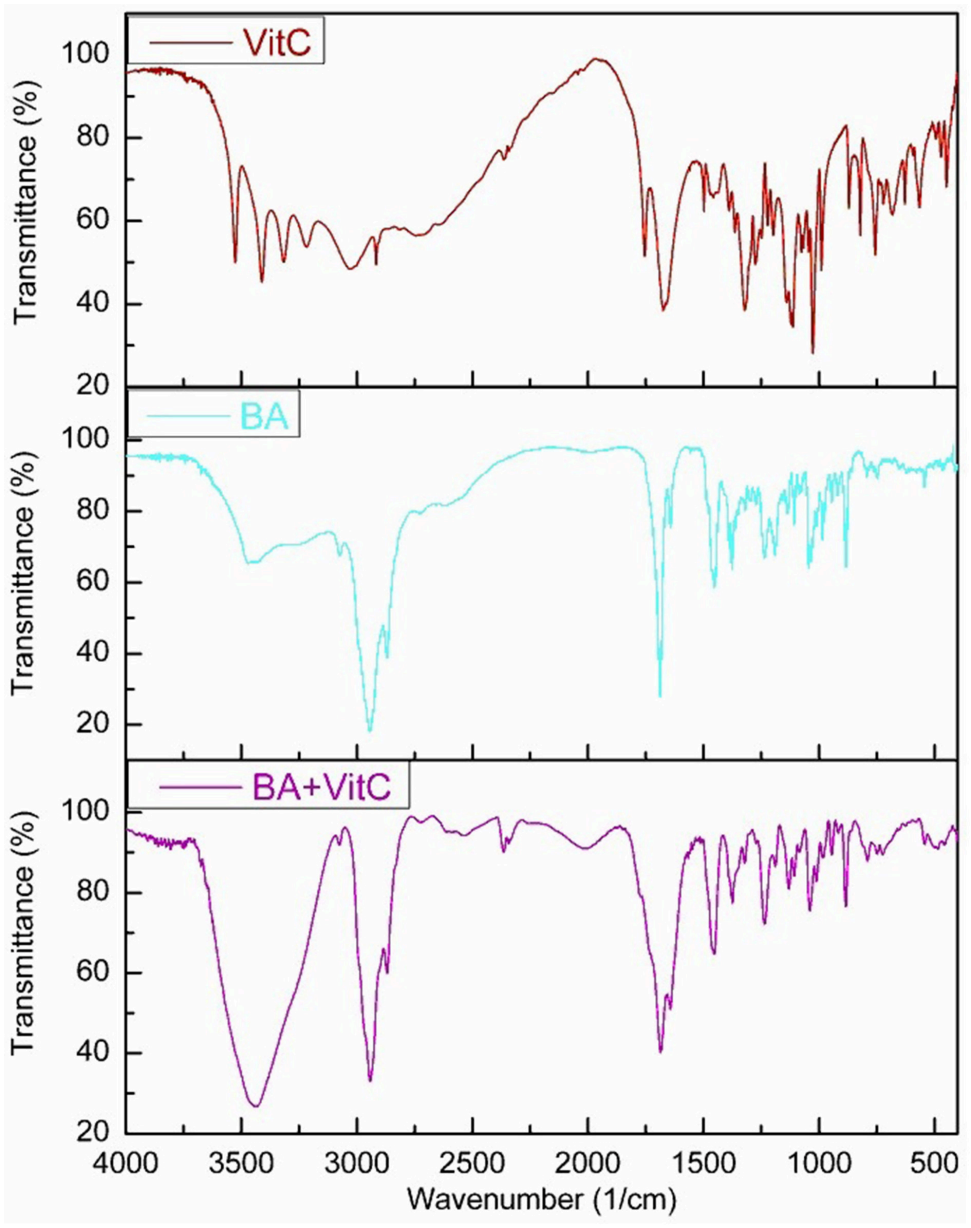
FTIR spectra for BA, VitC, and BA+VitC.

The FTIR spectra of the analyzed samples (Figure 5) reveal the binary adduct formation between the components, by the shifting of bands from the crystal to lower or higher wavenumbers, outside the range of ±5 cm^−1^. At high wavenumbers (spectral range 3,600–2,500 cm^−1^), the stretching of simple bonds such as O-H and C-H occurs. The broad band observed in the spectral range 3,600–3,400 cm^−1^ reveal the presence of intermolecular bonds, both in the case of pure BA, as in the case of the BA+VitC cocrystal. This observation leads to the conclusion that the formation of cocrystal occurs by hydrogen bonding.

The fingerprint region (spectral range 1,800–400 cm^−1^) contains numerous bands, due to the complex mode of vibration for both precursors (BA and VitC), as well as for the cocrystal. The modification of wavenumbers in this region is not easy to asses and is generally associated with bending and wagging (Schrader, 2008; Kovac-Besovic et al., 2009; Ahmad et al., 2010; Joshi et al., 2013; Wang et al., 2014; Stoler and Warner, 2015; Zhang et al., 2015; Yang et al., 2016). The experimental wavenumbers, expressed in cm^−1^, obtained in this study for the cocrystal of BA+VitC/VitC/BA present absorption bands at: 748/756/789 that could be assigned to C-C bending vibration; 883/-/884 assigned to = CH_2_ wagging vibration; 916/821/918 attributed to C-H and C-C bending vibration. The bands located at 982/869/983, 1010/989/1009, -/1078/1083, 1131/ 1143/ 1106 can be assigned to C-H, C-C and C-O bending vibrations. At 1235/1222/1239 is the band corresponding to C-H, C-C and O-H bending vibration. The bending vibration for C-H_2_ is located at -/1274/1298 and for C-CH_3_ is located at 1374/1388/1376. The bending vibration of C-H_2_ in the ring is present at 1453/1498/1450. The band present on the spectra at 1642/-/1643 correspond to C = C stretching vibration. At 1685/1679/1685 is the band that could be assigned to C = O stretching vibration in the ring. Vitamin C also presents a band at 1753 that can be attributed to C = O stretching vibration. C-H stretching vibration is present at 2869 for BA+VitC and at 2868 for BA. At 2942/2916/2940 is present the band that can be assigned to –CH_2_ stretching vibration. The band characteristic for the O-H stretching vibrations are 3075/3219/3203 and 3437/3315/3471.

### The Antioxidant Activity (AOA) Assessment

**Figure 6:**
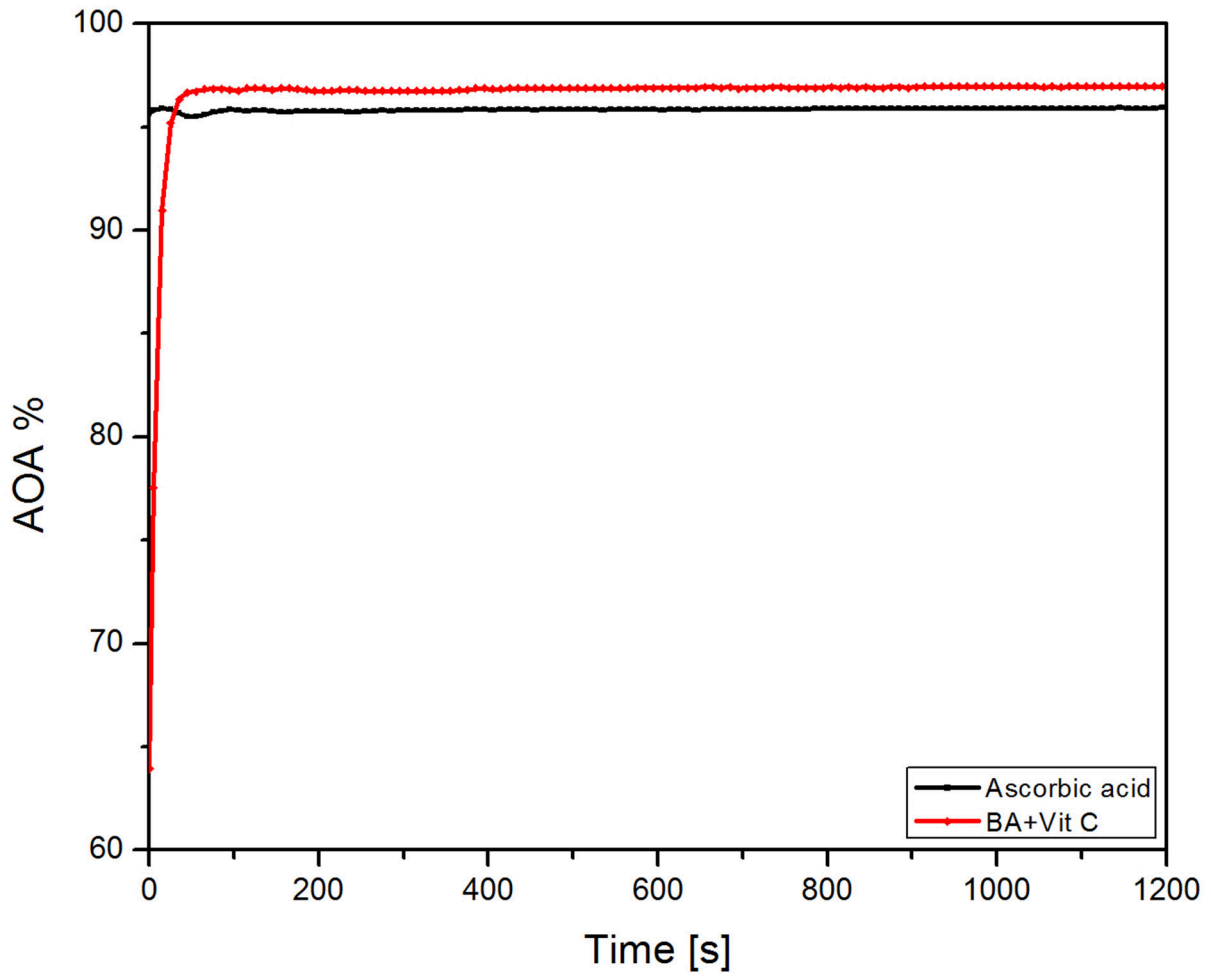
Recorded AOA of the BA+VitC cocrystal and ascorbic acid.

The AOA of the BA+VitC cocrystal and ascorbic acid, used as standard are exhibited in Figure 6. One can notice that the cocrystal shows as lightly increased AOA compared to that of pure ascorbic acid.

It can be assumed that the BA used in the preparation of the cocrystal has contributed to the reaction with DPPH thus leading to a higher AOA value compared to the one recorded for ascorbic acid. One can notice that values vary by 2% (ascorbic acid−95%, cocrystal−97%). The antioxidant activity of vitamin C was preserved in the 1:1 combination with BA and even increased by the presence of the triterpenic acid; however, the slight increase in antioxidant effect can be due to the intrinsic antioxidant activity of BA itself. Therefore, we can state at most that the antioxidant activities of both compounds was maintained in their cocrystal.

### *In vitro* Analysis

The cytotoxicity assessment of BA+VitC cocrystal solution was performed on healthy (HaCaT–immortalized human keratinocytes) and cancer (B16F10, B164A5–murine melanoma; MCF-7, MDA-MB-231–human breast carcinoma and HeLa–human cervix carcinoma) cell lines using three different concentrations−3, 10, and 30 μM by means of both MTT and Alamar blue assays. DMSO had no impact on cells viability, the inhibition values being similar with the ones obtained for control cells (unstimulated).

**Figure 7:**
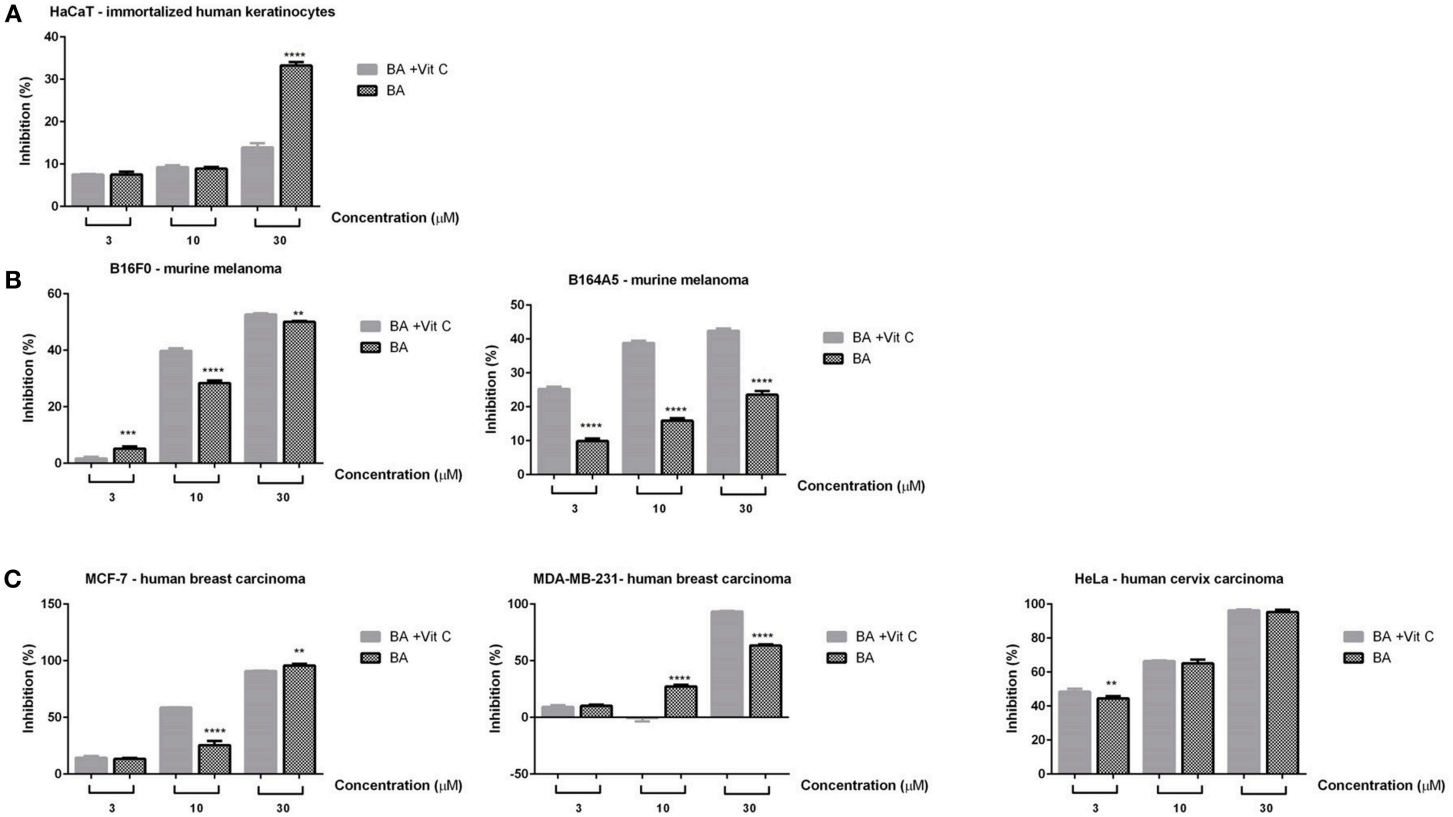
*In vitro* cytotoxicity assessment of BA and BA+VitC cocrystal (3, 10, and 30 μM) on immortalized human keratinocytes—HaCat **(A)**; murine melanoma cells–B16F0 and B164A5 **(B)**; breast–MCF-7 and MDA-MB-231–and cervical–HeLa cancer cells **(C)**, after 72 h stimulation, by the means of MTT assay. The results are expressed as inhibition index (%) related to control cells (unstimulated). The data represent the mean values ± SD of three independent experiments performed in triplicate. One-way ANOVA analysis was applied to determine the statistical differences followed by Tukey post-test (^**^p < 0.01; ^***^p < 0.001; ^****^p < 0.0001).

The stimulation of HaCaT cells with BA+VitC cocrystal at 3 and 10 μM for 72 h did not reveal a significant cytotoxic effect, the cellular inhibition being maintained below 10% (Figure 7A). The lack of toxic effects of betulinic acid on normal cells has been reported since the first research paper concerning its anti-melanoma activity (Pisha et al., 1995) although the respective paper analyzed its toxicity on *in vivo* animal models; therefore, betulinic acid was used as scaffold for various chemical derivatives with the purpose of preserving its good selectivity index (Waechter et al., 2017). Our study revealed however a rather significant cell inhibition (~40%) for BA alone at 30 μM that is strongly attenuated by the presence of vitamin C, being reduced at ~15%.

A significant cell inhibition was noted at the lowest concentration (3 μM) against HeLa cells (45%) for both BA and BA+VitC (Figure 7C); the inhibition increased with the applied dose for both samples thus presenting a dose-dependent profile: 70% cell inhibition at 10 μM and ~100% cell inhibition at 30 μM. The inhibitory effect was virtually equal for BA and BA+VitC at all concentrations.

Insignificant cell inhibition values were noted at 3 μM against MCF-7 and MDA-MB-231 cells (<10%) for BA and BA+VitC (Figure 7C). At 10 μM a 25% cell inhibition was reported for BA alone against MCF-7 cells while the inhibitory activity almost doubled for the cocrystal at the same concentration thus indicating a strong antiproliferative activity induced by the presence of VitC. When concentration increased at 30 μM a total cell inhibition was recorded for both BA and its cocrystal with VitC; therefore, it can be stated that the cell inhibition profile evolves in a dose-dependent manner.

Interestingly, the MDA-MB-231 cell line was moderately sensitive to 10 μM BA (25% cell inhibition) but did not react to the same concentration of BA+VitC cocrystal, when cell inhibition was practically null. The use of the highest tested concentration, 30 μM, induced an inhibitory activity of 60% for BA alone while in the presence of VitC cell inhibition reached 90% thus revealing a highly effective antiproliferative activity of the cocrystal.

Gao et al. reported in 2017 an important inhibition of the MDA-MB-231 cell line by various concentrations of betulinic acid ranging between 10 and 160 μM; however, in his study, cellular viability only decreased to around 50% when 40 μM BA was used, due to ultrastructural and morphological changes (Gao et al., 2018). In the current study, a similar cell inhibition was caused by a lower concentration of BA (30 μM) which significantly improves, reaching 90% cell inhibition, when the BA+VitC cocrystal was used. Our results are in line with the ones reported by Weber et al. who determined the IC_50_ value of betulinic acid against MDA-MB-231 cells as 21.9 μM thus revealing a strong cytotoxic effect (Weber et al., 2014). For HeLa cells, a 50% cellular viability was reported after 48 h stimulation with 30 μM BA, with a time- and dose-dependent inhibitory activity and an IC_50_ value of 30.42 ± 2.39 μM (Xu et al., 2017). The same group of authors reported in 2014 a 50% viability of HeLa cells after 48 h stimulation with 50 μM BA (Xu et al., 2014). By comparison, our results showed similar inhibition percentages at much lower concentrations (3–10 μM) while at 30 μM the cellular inhibition was almost complete both for BA and its cocrystal with VitC. An important antiproliferative activity was also previously reported by our group for BA against MCF-7 and HeLa cell lines (Soica et al., 2012).

In the case of murine melanoma cells–B16F10 (Figure 7B), the lowest concentration of both samples (3 μM) induced a negligible cell inhibition, whereas the higher concentrations−10 and 30 μM–exerted significant cytotoxic effects, the highest inhibition index being recorded after the 30 μM stimulation (around 50% for both samples); at 10 μM, a stronger cytotoxic activity was noted for BA+VitC cocrystal when compared to BA alone. The cell inhibition activity followed a dose-dependent profile. BA was previously reported as anti-melanoma agent (Pisha et al., 1995) and was revealed to increase in a synergistic manner the antitumor activity of vincristine in an *in vitro/in vivo* study on B16F10 cells (Sawada et al., 2004); BA caused the arrest of B16F10 melanoma cells in the G1 phase.

A visibly higher cytotoxic activity was recorded against B164A5 murine melanoma cells after stimulation with BA+VitC cocrystal as compared to BA alone, in all concentrations, respectively; the cell inhibition activity evolved in a dose-dependent manner. The highest inhibitory activity for BA alone (20%) was recorded at its highest used concentration (30 μM); in a similar manner, the strongest cell inhibition was exhibited by BA+VitC cocrystal at 30 μM but the value of cell inhibition was more than twice compared to BA alone (~45%).

Our group reported in 2014 the cytotoxic activity of BA against B164A5 cells leading to 50% cell viability at 10 mM; BA was solubilized by inclusion in octakis-[6-deoxy-6-(2-sulfanyl ethanesulfonic acid)]-γ-CD (Soica et al., 2014a). By comparison, the current study reports a similar citotoxic activity but using a much lower concentration of BA in the form of its VitC cocrystal (30 μM). The antimelanoma activity of BA was also confirmed using the murine B16/C57BL6 melanoma model (Soica et al., 2014a).

In terms of cytotoxic activity of vitamin C, the reported data is controversial. Most papers revealed the potentiation of the cytotoxic effects of various drugs following combination with vitamin C. Bober et al. reported in 2016 the up- and down-regulation of numerous proteins (229) by the combination of doxorubicin and vitamin C that resulted in the augmentation of the antiproliferative effect of doxorubicin (Bober et al., 2017). Guerriero et al. tested the cytotoxic effect of vitamin C, mitoxantrone, and their combination, respectively, against MCF-7and MDA-MB-231 breast cancer cells; they noticed a dose-dependent cell inhibition on both cell lines for both tested compounds while their combination provided a synergistic effect and allowed lower chemotherapic doses (Guerriero et al., 2014). The association of vitamin C with quercetine exerted antiproliferative effects against several breast cancer cell lines (MDA-MB-231, MCF-7, MDA-MB-468) through the induction of Nrf2-mediated oxidative stress in cancer cells in a synergistic manner as well (Mostafavi-Pour et al., 2017); the combination of vitamin C and quercetine with doxorubicin and paclitaxel caused a dramatical decrease of the IC_50_ of drugs and induced apoptosis in early stages (Ramezani et al., 2017). The administration of non-cytotoxic doses of vitamin C increased the chemotherapic response and apoptosis of HeLa cells treated with etoposide and cisplatin (Reddy et al., 2001). High concentrations of ascorbic acid (7–10 mM) administered intravenously were found to induce apoptosis of HeLa cells through the intrinsic and extrinsic pathways (Roberts et al., 2015); also, high doses of vitamin C inhibited energy metabolism in MCF-7 cancer cells through NAD depletion, thus inducing cell death (Uetaki et al., 2015). In counterpart, vitamin C was found to dose-dependently protect MCF-7 cancer cells against lipid peroxidation caused by tamoxifen thus causing a dose-dependent attenuation of cytotoxicity and diminishing the therapeutic response (Subramani et al., 2014).

Taking into account all these data, we can state that the cocrystallization of BA with vitamin C significantly improved the cytotoxic effect of BA against the tested tumor cell lines while preserving its good selectivity index. In part, we can assume that the increase of the biological activity of BA could be due to the intrinsic cytotoxic activity of vitamin C. However, the existing reports that incriminate vitamin C as cancer cell protector cast a shadow of doubt upon this assumption; in this regard, we may assume that vitamin C exhibited a protective effect on the MDA-MB-231 cancer cells when the 10 μM concentration was used, concentration at which BA alone caused a 25% cell inhibition. The mechanism behind this reported protective activity is yet to be investigated.

In the meantime, an important parameter in the investigation of poorly soluble drugs such as BA is solubility, parameter that can be greatly improved through cocrystallization (Qiao et al., 2011). As an example, the apparent solubility of vitamin K3 was greatly increased by cocrystallization with naphtoic acids and sulfamerazine (Zhu et al., 2015). Vitamin C was successfully employed as coformer in the cocrystallization of acyclovir for the purpose of improving the latter's water solubility and bioavailability (Meepripruk et al., 2016). Therefore, we presume that the experimental data reported here that revealed a very strong cytotoxic effect of BA+VitC cocrystals as compared to previously and currently reported data for BA alone are partially due to an increased water solubility of BA as a result of vitamin C cocrystallization; moreover, the presence of vitamin C as coformer may lead to an optimized bioavailability, subject that needs however further investigations.

## Data Availability

All data generated or analyzed during this study are included in this published article.

## Author Contributions

All authors conceived and designed the experiments. MN, RG, CS, AD, and RR conducted the experiments of obtaining the cocrystals and characterization by optical microscopy. MM performed DFT calculations. RA, RMR, MN, and MV performed XRD, DSC, and SEM experiments. MN, RG, and RMR conducted IR experiments. IZ, DC, CF, CD, and C-AD contributed with *in vitro* tests. EM and C-AD performed antioxidant activity. All authors analyzed the obtained data. All authors contributed with reagents, materials, and analysis instruments. All authors wrote the paper.

### Conflict of Interest Statement

The authors declare that the research was conducted in the absence of any commercial or financial relationships that could be construed as a potential conflict of interest.

## References

[B1] Adesanwo J. O. Makinde O. Obafemi C. (2013). Phytochemical analysis and antioxidant activity of methanol extract and betulinic acid isolated from the roots of tetracera potatoria. J. Pharm. Res. 6, 903–907. 10.1016/j.jopr.2013.09.003

[B2] Ahmad F. B. Ghaffari Moghaddam M. Basri M. Rahman M. (2010). Anticancer activity of 3- O -acylated betulinic acid derivatives obtained by enzymatic synthesis. Biosci. Biotechnol. Biochem. 74, 1025–1029. 10.1271/bbb.90917 20460723

[B3] Baltina L. A. Flekhter O. B. Nigmatullina L. R. Boreko E. I. Pavlova N. I. Nikolaeva S. N.. (2003). Lupane triterpenes and derivatives with antiviral activity. Bioorg. Med. Chem. Lett. 13, 3549–3552. 10.1016/S0960-894X(03)00714-5 14505668

[B4] Blois M. S. (1958). Antioxidant determinations by the use of a stable free radical. Nature 181, 1199–1200. 10.1038/1811199a0

[B5] Bober P. Alexovic M. Talian I. Tomkova Z. Viscorova Z. Benckova M.. (2017). Proteomic analysis of the vitamin C effect on the doxorubicin cytotoxicity in the MCF-7 breast cancer cell line. J. Cancer Res. Clin. Oncol. 143, 35–42. 10.1007/s00432-016-2259-4 27620743 PMC11819395

[B6] Chavda H. Patel C. Anand I. (2010). Biopharmaceutics classification system. Syst. Rev. Pharm. 1, 62–69. 10.4103/0975-8453.59514

[B7] Coricovac D.-E. Moaca E.-A. Pinzaru I. Citu C. Soica C. Mihali C.-V.. (2017). Biocompatible colloidal suspensions based on magnetic iron oxide nanoparticles: synthesis, characterization and toxicological profile. Front. Pharmacol. 8:154. 10.3389/fphar.2017.00154 28400730 PMC5368253

[B8] Das J. Samadder A. Das S. Paul A. Khuda-Bukhsh A. R. (2016). Nanopharmaceutical approach for enhanced anti-cancer activity of betulinic acid in lung-cancer treatment via activation of PARP: interaction with dna as a target: -anti-cancer potential of nano-betulinic acid in lung cancer. J. Pharmacopuncture 19, 37–44. 10.3831/KPI.2016.19.005 27280048 PMC4887750

[B9] Dehelean C. A. Feflea S. Gheorgheosu D. Ganta S. Cimpean A. M. Muntean D.. (2013). Anti-angiogenic and anti-cancer evaluation of betulin nanoemulsion in chicken chorioallantoic membrane and skin carcinoma in Balb/c mice. J. Biomed. Nanotechnol. 9, 577–589. 10.1166/jbn.2013.1563 23621016

[B10] Du Y. Cai Q. Xue J. Zhang Q. (2016). Raman and terahertz spectroscopic investigation of cocrystal formation involving antibiotic nitrofurantoin drug and coformer 4-aminobenzoic acid. Crystals 6:164 10.3390/cryst6120164

[B11] Faujan N. Alitheen N. Yeap S. K. Ali A. H. Muhajir A. (2009). Cytotoxic effect of betulinic acid and betulinic acid acetate isolated from Melaleuca cajuput on human myeloid leukemia (HL-60) cell line. African J. Biotechnol. 9, 6387–6396.

[B12] Fulda S. Kroemer G. (2009). Targeting mitochondrial apoptosis by betulinic acid in human cancers. Drug Discov. Today 14, 885–890. 10.1016/j.drudis.2009.05.015 19520182

[B13] Gadade D. Pekamwar S. Lahoti S. Patni S. D. (2016). Cocrystallization of etodolac: prediction of cocrystallization, synthesis, solid state characterization and *in vitro* drug release. Marmara Pharm. J. 21, 78–88. 10.12991/marupj.259884

[B14] Gao Y. Ma Q. Ma Y. B. Ding L. Xu X. L. Wei D. F.. (2018). Betulinic acid induces apoptosis and ultrastructural changes in MDA-MB-231 breast cancer cells. Ultrastruct. Pathol. 42, 49–54. 10.1080/01913123.2017.1383548 29192840

[B15] Godugu C. Patel A. R. Doddapaneni R. Somagoni J. Singh M. (2014). Approaches to improve the oral bioavailability and effects of novel anticancer drugs berberine and betulinic acid. PLoS ONE 9:e89919. 10.1371/journal.pone.0089919 24614362 PMC3948684

[B16] Grecu T. Hunter C. A. Gardiner E. J. Mccabe J. F. (2013). Validation of a computational cocrystal prediction tool: comparison of virtual and experimental cocrystal screening results. Cryst. Growth Des. 14, 165–171. 10.1021/cg401339v

[B17] Guerriero E. Sorice A. Capone F. Napolitano V. Colonna G. Storti G.. (2014). Vitamin C effect on mitoxantrone-induced cytotoxicity in human breast cancer cell lines. PLoS ONE 9:e115287. 10.1371/journal.pone.0115287 25531443 PMC4274052

[B18] Jain S. Patel N. Lin S. (2015). Solubility and dissolution enhancement strategies: current understanding and recent trends. Drug Dev. Ind. Pharm. 41, 875–887. 10.3109/03639045.2014.971027 25342479

[B19] Joshi H. Kumar Saxena G. Singh V. Arya E. Singh R. (2013). Phytochemical investigation, isolation and characterization of betulin from bark of *Betula utilis*. J. Pharmacogn. Phytochem. 2, 145–151.

[B20] Karagianni A. Malamatari M. (2018). Pharmaceutical cocrystals: new solid phase modification approaches for the formulation of APIs. Pharmaceutics 10, 1–30. 10.3390/pharmaceutics10010018 29370068 PMC5874831

[B21] Kavuru P. Aboarayes D. Arora K. Clarke H. Kennedy A. Marshall L. (2010). Hierarchy of supramolecular synthons: persistent hydrogen bonds between carboxylates and weakly acidic hydroxyl moieties in cocrystals of zwitterions. Cryst. Growth Des 10:3568 10.1021/cg100484a

[B22] Kovac-Besović E. E. Duric K. Kalodera Z. Sofić E. (2009). Identification and isolation of pharmacologically active triterpenes in betuale cortex, betula pendula roth., Betulaceae. Bosn. J. Basic Med. Sci. 9, 31–38. 10.17305/bjbms.2009.2853 19284392 PMC5645545

[B23] Leyssens T. Tumanova N. Robeyns K. Nadine C. Veesler S. (2014). Solution cocrystallization, an effective tool to explore the variety of cocrystal systems: caffeine/dicarboxylic acid cocrystals. CrystEngComm 16, 9603–9611. 10.1039/C4CE01495B

[B24] Lomkova E. A. Chytil P. Janouskova O. Mueller T. Lucas H. Filippov S.. (2016). Biodegradable micellar HPMA-based polymer-drug conjugates with betulinic acid for passive tumor targeting. Biomacromolecules 17, 3493–3507. 10.1021/acs.biomac.6b00947 27636143

[B25] Meepripruk M. Bumee R. Somphon W. Toh P. (2016). Crystal growth and physical characterization of acyclovir crystallized with ascorbic acid and zinc chloride. J. Life Sci. Technol. 4, 56–59. 10.18178/jolst.4.2.56-59

[B26] Mosmann T. (1984). Rapid colorimetric assay for cellular growth and survival - application to proliferation and cyto-toxicity assays. J. Immunol. Methods 65, 55–63. 10.1016/0022-1759(83)90303-4 6606682

[B27] Mostafavi-Pour Z. Ramezani F. Keshavarzi F. Samadi N. (2017). The role of quercetin and vitamin C in Nrf2-dependent oxidative stress production in breast cancer cells. Oncol. Lett. 13, 1965–1973. 10.3892/ol.2017.5619 28454351 PMC5403368

[B28] Mukherjee R. Jaggi M. Rajendran P. Siddiqui M. J. K. Srivastava S. (2004). Betulinic acid and its derivatives as anti-angiogenic agents. Bioorg. Med. Chem. Lett. 14, 2181–2184. 10.1016/j.bmcl.2004.02.044 15081004

[B29] Mullauer F. B. van Bloois L. Daalhuisen J. B. Ten Brink M. S. Storm G. Medema J. P.. (2011). Betulinic acid delivered in liposomes reduces growth of human lung and colon cancers in mice without causing systemic toxicity. Anticancer Drugs 22, 223–233. 10.1097/CAD.0b013e3283421035 21263311

[B30] Musumeci D. Hunter C. A. Prohens R. Mccabe J. F. (2011). Virtual cocrystal screening. Chem. Sci. 2, 883–890. 10.1039/c0sc00555j

[B31] Pathak C. D. Savjani K. T. Gajjar A. Savjani J. (2013). Cocrystal formation of paracetamol with indomethacin and mefenamic acid: an efficient approach to enhance solubility. Int. J. Pharm. Pharm. Sci. 5, 414–419.

[B32] Peng J. Lv Y. C. He P. P. Tang Y. Y. Xie W. Liu X. Y.. (2015). Betulinic acid downregulates expression of oxidative stress-induced lipoprotein lipase via the PKC/ERK/c-Fos pathway in RAW264.7 macrophages. Biochimie 119, 192–203. 10.1016/j.biochi.2015.10.020 26542288

[B33] Pisha E. Chai H. S. Lee I. E. Chagwedera T.. (1995). Discovery of betulinic acid as a selective inhibitor of human melanoma that functions by induction of apoptosis. Nat. Med. 1, 1046–1051. 10.1038/nm1095-1046 7489361

[B34] Prasad R. V. Rakesh M. G. Jyotsna R. M. Mangesh S. T. Anita P. S. Mayur P. K. (2012). Pharmaceutical cocrystallization: a review. Pharm. Cocrystallization A Rev. 1, 725–736.

[B35] Qiao N. Li M. Schlindwein W. Malek N. Davies A. Trappitt G. (2011). Pharmaceutical cocrystals: an overview. Int. J. Pharm. 419, 1–11. 10.1016/j.ijpharm.2011.07.037 21827842

[B36] Ramezani F. Samadi N. Mostafavi-Pour Z. (2017). Sequential therapy of breast cancer cell lines with vitamin C and quercetin improves the efficacy of chemotherapeutic drugs. Nutr. Cancer 69, 881–891. 10.1080/01635581.2017.1339813 28742385

[B37] Reddy V. G. Khanna N. Singh N. (2001). Vitamin C augments chemotherapeutic response of cervical carcinoma HeLa cells by stabilizing P53. Biochem. Biophys. Res. Commun. 282, 409–415. 10.1006/bbrc.2001.4593 11401473

[B38] Roberts B. M. Fullerton D. R. Elliott S. L. (2015). High concentrations of L-ascorbic acid (Vitamin C) induces apoptosis in a human cervical cancer cell line (HeLa) through the intrinsic and extrinsic pathways. Bios 86, 134–143. 10.1893/BIOS-D-14-00019.1

[B39] Sanphui P. Devi V. K. Clara D. Malviya N. Ganguly S. Desiraju G. R. (2015). Cocrystals of hydrochlorothiazide: solubility and diffusion/permeability enhancements through drug-coformer interactions. Mol. Pharm. 12, 1615–1622. 10.1021/acs.molpharmaceut.5b00020 25800383

[B40] Sawada N. Kataoka K. Kondo K. Arimochi H. Fujino H. Takahashi Y.. (2004). Betulinic acid augments the inhibitory effects of vincristine on growth and lung metastasis of B16F10 melanoma cells in mice. Br. J. Cancer 90, 1672–1678. 10.1038/sj.bjc.6601746 15083202 PMC2409700

[B41] Schrader B. (2008). Infrared and Raman Spectroscopy: Methods and Applications. Wiley 10.1002/9783527615438

[B42] Schultheiss N. Newman A. (2009). Pharmaceutical cocrystals and their physicochemical properties. Cryst. Growth Des. 9, 2950–2967. 10.1021/cg900129f 19503732 PMC2690398

[B43] Sekhon B. S. (2012). Drug-drug co-crystals. DARU J. Pharm. Sci. 20, 20–45. 10.1186/2008-2231-20-45 23351300 PMC3555774

[B44] Soica C. Danciu C. Savoiu-Balint G. Borcan F. Ambrus R. Zupko I.. (2014a). Betulinic acid in complex with a gamma-cyclodextrin derivative decreases proliferation and *in vivo* tumor development of non-metastatic and metastatic B164A5 cells. Int. J. Mol. Sci. 15, 8235–8255. 10.3390/ijms15058235 24821543 PMC4057729

[B45] Soica C. Oprean C. Borcan F. Danciu C. Trandafirescu C. Coricovac D.. (2014b). The synergistic biologic activity of oleanolic and ursolic acids in complex with hydroxypropyl-?-cyclodextrin. Molecules 19, 4924–4940. 10.3390/molecules19044924 24747649 PMC6271422

[B46] Soica C. M. Dehelean C. A. Peev C. Aluas M. Zupkó I. Kása P.. (2012). Physico-chemical comparison of betulinic acid, betulin and birch bark extract and *in vitro* investigation of their cytotoxic effects towards skin epidermoid carcinoma (A431), breast carcinoma (MCF7) and cervix adenocarcinoma (HeLa) cell lines. Nat. Prod. Res. 26, 968–974. 10.1080/14786419.2010.545352 21598174

[B47] Stoler E. Warner J. (2015). Non-Covalent derivatives: cocrystals and eutectics. Molecules 20, 14833–14848. 10.3390/molecules200814833 26287141 PMC6332263

[B48] Subramani T. Yeap S. K. Ho W. Y. Ho C. L. Omar A. R. Aziz S. A.. (2014). Vitamin C suppresses cell death in MCF-7 human breast cancer cells induced by tamoxifen. J. Cell. Mol. Med. 18, 305–313. 10.1111/jcmm.12188 24266867 PMC3930417

[B49] Sun C. (2012). Cocrystallization for successful drug delivery. Expert Opin. Drug Deliv. 10, 201–213. 10.1517/17425247.2013.747508 23256822

[B50] Tan J. Govindarajan K. Arulselvan P. Fakurazi S. Hussein M. (2014). Sustained release and cytotoxicity evaluation of carbon nanotube-mediated drug delivery system for betulinic acid. J. Nanomater. 2014, 1–11. 10.1155/2014/862148

[B51] Tomaszewska I. Karki S. Shur J. Price R. Fotaki N. (2013). Pharmaceutical characterisation and evaluation of cocrystals: importance of *in vitro* dissolution conditions and type of coformer. Int. J. Pharm. 453, 380–388. 10.1016/j.ijpharm.2013.05.048 23727143

[B52] Uetaki M. Tabata S. Nakasuka F. Soga T. Tomita M. (2015). Metabolomic alterations in human cancer cells by Vitamin C-induced oxidative stress. Sci. Rep. 5:13896. 10.1038/srep13896 26350063 PMC4563566

[B53] Venugopalaiah P. Sravanthi D. Gobinath M. Kumar B. Dinesh R. (2016). Pharmaceutical co-crystals - an approach to increase solubility and bioavailability. IJPIB 1, 63–70.

[B54] Waechter F. da Silva G. N. S. Willig J. B. de Oliveira C. B. Vieira B. D. Trivella D. B. B.. (2017). Design, synthesis and biological evaluation of betulinic acid derivatives as new antitumor agents for leukemia. Anticancer. Agents Med. Chem. 17, 1777–1785. 10.2174/1871521409666170412143638 28403779

[B55] Wang X. Gong N. Yang S. Du G. Lu Y. (2014). Studies on solvatomorphism of betulinic acid. J. Pharm. Sci. 103, 2696–2703. 10.1002/jps.23853 24752825

[B56] Weber D. Zhang M. Zhuang P. Zhang Y. Wheat J. Currie G.. (2014). The efficacy of betulinic acid in triple-negative breast cancer. SAGE Open Med. 2. 10.1177/2050312114551974 26770742 PMC4607226

[B57] Xu T. Pang Q. Wang Y. Yan X. (2017). Betulinic acid induces apoptosis by regulating PI3K/Akt signaling and mitochondrial pathways in human cervical cancer cells. Int. J. Mol. Med. 40, 1669–1678. 10.3892/ijmm.2017.3163 29039440 PMC5716432

[B58] Xu T. Pang Q. Zhou D. Zhang A. Luo S. Wang Y.. (2014). Proteomic investigation into betulinic acid-induced apoptosis of human cervical cancer HeLa cells. PLoS ONE 9:e105768. 10.1371/journal.pone.0105768 25148076 PMC4141803

[B59] Yamashita H. Hirakura Y. Yuda M. Teramura T. Terada K. (2013). Detection of cocrystal formation based on binary phase diagrams using thermal analysis. Pharm. Res. 30, 70–80. 10.1007/s11095-012-0850-1 22907418

[B60] Yang D. Gong N. Zhang L. Lu Y. Du G. (2016). Structural and computational study of four new solvatomorphs of betulin: a combined X-ray, Hirshfeld surface, and thermal analysis. J. Pharm. Sci. 106, 826–834. 10.1016/j.xphs.2016.11.004 27989367

[B61] Zaworotko M. Clarke H. Kapildev A. Kavuru P. Shytle R. D. Pujari T. (2008). Nutraceutical co-crystal compositions. WO patent number 2008/153945A2.

[B62] Zhang Z.-H. Zeng X.-A. Brennan C. S. Brennan M. Han Z. Xiong X.-Y. (2015). Effects of pulsed electric fields (PEF) on vitamin C and its antioxidant properties. Int. J. Mol. Sci. 16, 24159–24173. 10.3390/ijms161024159 26473846 PMC4632744

[B63] Zhu B. Wang J.-R. Zhang Q. Mei X. (2015). Improving dissolution and photostability of vitamin K3 via cocrystallization with naphthoic acids and sulfamerazine. Cryst. Growth Des. 16, 483–492. 10.1021/acs.cgd.5b01491

[B64] Ziegler H. L. Franzyk H. Sairafianpour M. Tabatabai M. Tehrani M. D. Bagherzadeh K.. (2004). Erythrocyte membrane modifying agents and the inhibition of Plasmodium falciparum growth: structure-activity relationships for betulinic acid analogues. Bioorg. Med. Chem. 12, 119–127. 10.1016/j.bmc.2003.10.010 14697777

